# Prevalence of pain in community-dwelling older adults with hypertension in the United States

**DOI:** 10.1038/s41598-022-12331-0

**Published:** 2022-05-19

**Authors:** Chao-Yi Li, Wei-Cheng Lin, Ching-Yen Lu, Yu Shan Chung, Yu-Chen Cheng

**Affiliations:** 1 School of Medicine, College of Medicine, Fu Jen Catholic University, New Taipei City, Taiwan, ROC; 2 Department of Neurology, Fu Jen Catholic University Hospital, Fu Jen Catholic University, No. 69, Guizi Road, Taishan District, New Taipei City, Taiwan, ROC

**Keywords:** Epidemiology, Pain, Hypertension

## Abstract

Hypertension and pain are both prevalent conditions in the older adult population. We aimed to report the prevalence of pain discomforts and investigated the association between hypertension and pain discomforts among older adults in the United States. Data from the 2011 National Health and Aging Trends Study were analyzed. In-person interviews were conducted in 7601 adults ages ≥ 65 years. Prevalence of bothersome pain, activity-limiting pain, locations of pain and usage of pain medicine were evaluated. Demographics, comorbidities, and other covariates were compared between older adults with hypertension and those without. Multivariate regression was further performed to yield adjusted odd ratios. Among 6825 older adults, 4533 of them had a history of hypertension while 2272 of them had not. Prevalence of bothersome pain (57.12% versus 44.81%, p < 0.001) and activity-limiting pain (56.21% versus 46.12%, p < 0.001) were significantly higher in the hypertension group. After adjusting for all covariates, hypertension demonstrated a significant association with activity-limiting pain (OR 1.63, 95% CI 1.06 to 2.52, p = 0.02). In conclusion, pain was more prevalent in older Americans with hypertension. The positive association between hypertension and pain suggested that routine pain assessment and proper treatment would be required to improve the function and quality of life among older adults especially with hypertension.

## Introduction

Hypertension, which is one of the most important public health issues, usually leads to serious cardiovascular outcomes, such as premature death, coronary heart disease, heart failure, and cerebrovascular disease^1^. The prevalence of hypertension increases with age; while the overall prevalence of hypertension was 45.4% in the United States, the prevalence increased from 22.4% among young adults (aged 18–39 years old) to 74.5% among older adults (aged ≥ 60 years old)^2^. Given that life expectancy has extended over the past decades, the growth of the older population has increased the effects of hypertension and its comorbidities. Pain is also a prevalent health condition in the United States, with chronic pain affecting approximately 20% (50.0 millions) of American adults^3^. The prevalence of chronic pain increases with age and is doubled among older adults (aged 25–44: 13.2 vs. aged 65–84: 27.6%). Chronic pain not only influences quality of life^4^, but also causes severe adverse outcomes, such as disability and mortality^5,6^ however, little is known about the experience of pain in community-dwelling older adults with hypertension in the United States.

The relationship between hypertension and pain is intertwined but not well understood. Previous population-based studies have demonstrated an inverse relationship between hypertension and migraine, low back pain, osteoarthritis, and chronic musculoskeletal complaints^7–9^. In contrast, an increased risk of hypertension is observed in patients with chronic pain relative to that in a pain-free population^10–12^. The direction of the relationship between hypertension and pain may be influenced by sex, antihypertensive medication, and chronification of pain^13,14^. While the inverse relationship occurs with short-term pain stimuli among normotensive individuals, the long-term relationship may be the opposite^14^.

Understanding the prevalence of pain, demographics, comorbidities, and psychological and physical symptoms in patients with hypertension will provide an important view for better understanding and further employment of treatments for patients with both hypertension and pain. While the prevalence of hypertension has been examined in patients with pain disorders in the community and tertiary pain centers^11–13^, few studies have focused on the appropriate assessment of pain burden among older adults with hypertension. The present study aimed to provide a robust viewpoint on the prevalence of pain discomfort among community-dwelling older adults with hypertension in a large-scale cross-sectional study using the National Health and Aging Trends Study (NHATS) cohort in the United States.

## Methods

### Material and methods

#### Study population

We acquired the 2011 questionnaire from the NHATS dataset. The NHATS, conducted by Johns Hopkins Bloomberg School of Public Health, is a longitudinal survey that focuses on health, functional, and social outcomes among nationally representative Medicare beneficiaries aged ≥ 65 years in the United States. The annual structured interview in the NHATS contains demographic data, socioeconomic information, health conditions, and healthcare usage of the participants. The informed consent of study participants and the design and sampling methodology of NHATS have been reported previously^15^. The NHATS study was approved by the Johns Hopkins School of Public Health Institutional Review Board under the HIPAA privacy rule and the participants provided written informed consent. All study protocols were conducted in compliance with the principles of the Declaration of Helsinki guidelines. Since the NHATS data were de-identified, this secondary data analysis study qualified an exemption and was approved by the Institutional Review Board of the Fu Jen Catholic University Institutional Review Board. This study follows the Strengthening the Reporting of Observational Studies in Epidemiology (STROBE) reporting guidelines. We confirmed that all methods were performed in accordance with relevant guidelines and regulations.

We included participants who were enrolled in the NHATS cohort at the first-round interview in 2011 (n = 8245), and excluded those with a history of dementia (n = 1420). The final sample consisted of 6825 Medicare beneficiaries aged ≥ 65 years. Participants were asked if they had been told that they had high blood pressure by doctors before the baseline visit in 2011. The study population was divided into two groups: a hypertension group (n = 4553) and a control group (n = 2272).

### Measures

#### Pain

Pain was assessed through interviews. The main pain measures included bothersome pain and activity-limiting pain. Participants were asked whether they had been bothered by pain in the previous month. Those who answered “yes” were asked whether their pain had limited their activities. Other pain measures included the location of pain and frequency of taking medicine for pain. Participants were required to specify the location of pain in the last month according to the anatomic site: back, hips, knees, legs, feet, hands, wrists, arms, shoulders, stomach, head, neck, and others. Participants were also asked how often they took medicine for pain in five categories: every day (7 days per week), most days (5–6 days per week), some days (2–4 days per week), rarely (once a week or less), and never (0 days per week).

#### Hypertension

Hypertension was determined using self-reported diagnostic information. Participants were asked if they were told by doctors that they had high blood pressure or hypertension.

#### Covariates

Other measures were all collected during the survey in 2011 and classified into six categories, including demographic information, comorbidities, psychological symptoms, physical symptoms, physical capacity, and sleep symptoms.

#### Basic demographics

Demographic information included sex, age, race, marital status, education level, total income, residential care status, and body mass index (BMI). Age was categorized into six groups: 65–69, 70–74, 75–79, 80–84, 85–89, and ≥ 90 years old. Self-identified race and ethnicity were divided into four groups: white, non-Hispanic; black, non-Hispanic; Hispanic; and other, non-Hispanic. Relationship status was divided into having a partner (married or living with a partner) and not having a partner. Education level was dichotomized into two groups: (1) less than high school and (2) high school graduate or above. Due to the missing total income data, we used the five input values of total annual income created by the NHATS^16^. The average of the five input values was calculated for individuals with missing data. We then categorized total income into two groups based on the median of the overall data, which was US$28,000: (1) < $28,000 and (2) ≥ $28,000. Residential care status was divided into living in the community and living in non-nursing home residential care. BMI was calculated as weight (kg)/height squared (m^2^). BMI ranges were classified into four groups according to the World Health Organization: underweight (BMI < 18.5 kg/m^2^), normal (BMI 18.5–24.9 kg/m^2^), overweight (BMI: 25–29.9 kg/m^2^), and obese (BMI > 30 kg/m^2^).

#### Comorbidities

Participants were asked if they had been told by doctors that they had previously had any of the following: heart attack, heart disease, arthritis, osteoporosis, diabetes, lung disease, stroke, cancer, and hip or other fracture.

#### Psychological symptoms

Psychological symptoms included depression and anxiety. The Patient Health Questionnaire (PHQ)-2 and General Anxiety Disorder (GAD)-2 scores were calculated to represent these two symptoms, respectively. PHQ-2 and GAD-2 scores ≥ 3 have been shown to be related to a greater risk of depression^17^ and anxiety^18^.

#### Physical symptoms

Physical symptoms included activity-limiting dyspnea and activity-limiting fatigue. Participants were asked if they had breathing problems, low energy, or exhaustion that limited their activities.

#### Physical capacity

Physical capacity was assessed using a short physical performance battery (SPPB). The SPPB score evaluated lower extremity function through a standing balance test, gait speed test, and ability to rise from a chair^19^.The sum of the scores of the three tests ranges 0–12. The SPPB has been recognized as a reliable and predictable measure of mobility and disability levels^20^. We categorized the results into three groups: low capacity (SPPB 0–4), intermediate capacity (SPPB 5–8), and high capacity (SPPB 9–12).

#### Sleep symptoms

Sleep symptoms included sleep difficulties and medication use. Participants were asked how often they had sleep problems, such as taking over 30 min to fall asleep in the last month, how often they had trouble falling asleep at night in the last month, and how often they took medication for sleep in the last month. The answers were classified into five groups: every night (7 days a week); most nights (5–6 days a week); some nights (2–3 days a week); rarely (once a week or less) and refuse to answer, never, and unknown. Sleep difficulty was defined based on the following factors: taking over 30 min to fall asleep and having trouble falling asleep at least 2 days per week in the last month. Sleep medication was defined as taking medication for at least 2 days per week in the last month.

### Statistical analysis

The demographics and covariates of the hypertension group and control group are summarized as means ± standard deviation or frequency (percentage) and analyzed using the *t*-test and *χ*^2^ statistics. Linear mixed models were used to examine the associations of bothersome pain and activity-limiting pain in patients with a history of hypertension (hypertension group) and those without any history of hypertension (control group), adjusting for basic demographic factors (sex, age, race, marital status, education level, total income, residential care status, and BMI range), comorbidities (heart attack, heart disease, arthritis, osteoporosis, diabetes, hypertension, lung disease, stroke, cancer, hip fracture, and other fractures), psychological symptoms (depression and anxiety), physical symptoms (activity-limiting dyspnea, activity-limiting fatigue), physical capacity (low capacity, intermediate capacity, and high capacity), and sleep symptoms (sleep difficulty and sleep medicine).

Stepwise regression was performed for model building. Hypertension was maintained at every step because it was the exposure of primary interest. After checking for collinearity, covariate variables that were suggestive of a possible bivariate association (i.e., *P* < 0.25) were included in the initial multivariable models. Backward elimination was used to analyze the proper multivariable models. The covariates remained in the candidate list with the selection criteria set at *P* < 0.10. For the final model building, *P* < 0.05 was set as the selection criterion. Stata software, version 14 (Stata Corp., College Station, TX, USA) was used for all statistical analyses.

## Results

The study population consisted of 6825 older adults, of whom 4553 (66.71%) had a history of hypertension and 2272 (33.29%) did not. Of all participants, 37.78% were aged ≥ 80 years, 57.89% were female, and 95.18% were lived in the community. Overall, 3625 (53.01%) participants reported bothersome pain, while 1930 (28.28%) reported activity-limiting pain. Table 1 summarizes the demographic characteristics of the hypertension and control groups. In comparison with the control group, the hypertension group tended to be older and more likely to be obese, female, and non-Hispanic black, had lower total income, lower educational level, and a lower percentage of individuals who were married or living with a partner. Older adults with hypertension also tended to have more comorbidities, including heart attacks, heart disease, arthritis, osteoporosis, diabetes, lung disease, stroke, and cancer. Moreover, those with hypertension were likely to have more psychological symptoms (depression and anxiety), and more activity-limiting fatigue. Lower physical capacity, more sleep difficulty, and more medication taken for sleep were also noted in older individuals with hypertension. The hypertension and control groups did not differ significantly in terms of the following demographic characteristics: residential care status, history of hip and other fractures, and symptoms of activity-limiting dyspnea.

**Table 1:** Basic demographics.

	Hypertension (N = 4553)	Control (N = 2272)	*p*–value
**Demographic**			
Gender (female, %)	2731 (59.98%)	1220 (53.70%)	< 0.001
Age (n, %)			< 0.001
65–69	833 (18.30%)	529 (23.28%)	
70–74	1014 (22.27%)	490 (21.57%)	
75–79	955 (20.98%)	425 (18.71%)	
80–84	940 (20.65%)	389 (17.12%)	
85–89	519 (11.40%)	251 (11.05%)	
≥ 90	292 (6.41%)	188 (8.27%)	
Race/Ethnicity			< 0.001
White, non-Hispanic	2997(66.45%)	1761 (78.44%)	
Black, non-Hispanic	1164(25.81%)	287 (12.78%)	
Hispanic	108 (2.39%)	62 (2.76%)	
Others	241 (5.34%)	135(6.01%)	
Education (≥ High school)	3286 (72.78%)	1782 (79.31%)	< 0.001
Marital status			
Married/live with a partner	2257 (49.63%)	1248 (54.98%)	< 0.001
Residential care status			0.859
Community	4335 (95.21%)	2161 (95.11%)	
Non–nursing home residential care	218 (4.79%)	111 (4.89%)	
BMI range			
Underweight (< 18.5)	71 (1.56%)	68 (3.00%)	< 0.001
Normal (18.5–24.9)	1222 (26.84%)	873 (38.42%)	< 0.001
Overweight (25.0–29.9)	1613 (35.43%)	809 (35.61%)	0.863
Obese (≥ 30.0)	1443 (31.69%)	410 (18.05%)	< 0.001
Income (≥ 28,000 USD)	1242(48.35%)	707 (56.02%)	< 0.001
**Comorbidity**			
Heart attack	754 (16.58%)	253 (11.15%)	< 0.001
Heart disease	953 (20.96%)	251 (11.06%)	< 0.001
Arthritis	2716 (59.77%)	1036 (45.66%)	< 0.001
Osteoporosis	932 (20.53%)	407 (17.96%)	0.012
Diabetes	1398 (30.71%)	320 (14.09%)	< 0.001
Lung disease	751 (16.51%)	274 (12.07%)	< 0.001
Stroke	534 (11.74%)	150 (6.61%)	< 0.001
Cancer	1231 (27.05%)	521 (22.93%)	< 0.001
Hip fracture	204 (4.48%)	82 (3.61%)	0.092
Other fracture	925 (20.33%)	418 (18.41%)	0.060
**Psychological symptoms**			
Depression	2178 (47.84%)	832 (36.62%)	< 0.001
Anxiety	2117 (46.5%)	830 (36.53%)	< 0.001
**Physical symptoms**			
Dyspnea limits activity	542 (49.63%)	163 (47.94%)	0.586
Fatigue limits activity	1476 (66.7%)	474 (59.55%)	< 0.001
**Physical capacity**			
Low capacity (SPPB 0–4)	1281 (28.14%)	489 (21.52%)	< 0.001
Middle capacity (SPPB 5–8)	1656 (36.37%)	726 (31.95%)	< 0.001
High capacity (SPPB 9–12)	1021 (22.42%)	796 (35.04%)	< 0.001
**Sleep symptoms**			
Sleep difficulty	2757 (60.55%)	1198 (52.73%)	< 0.001
Sleep medication	1013 (22.28%)	393 (17.35%)	< 0.001

*BMI* body mass index, *SPPB* short physical performance battery.

**Figure 1:**
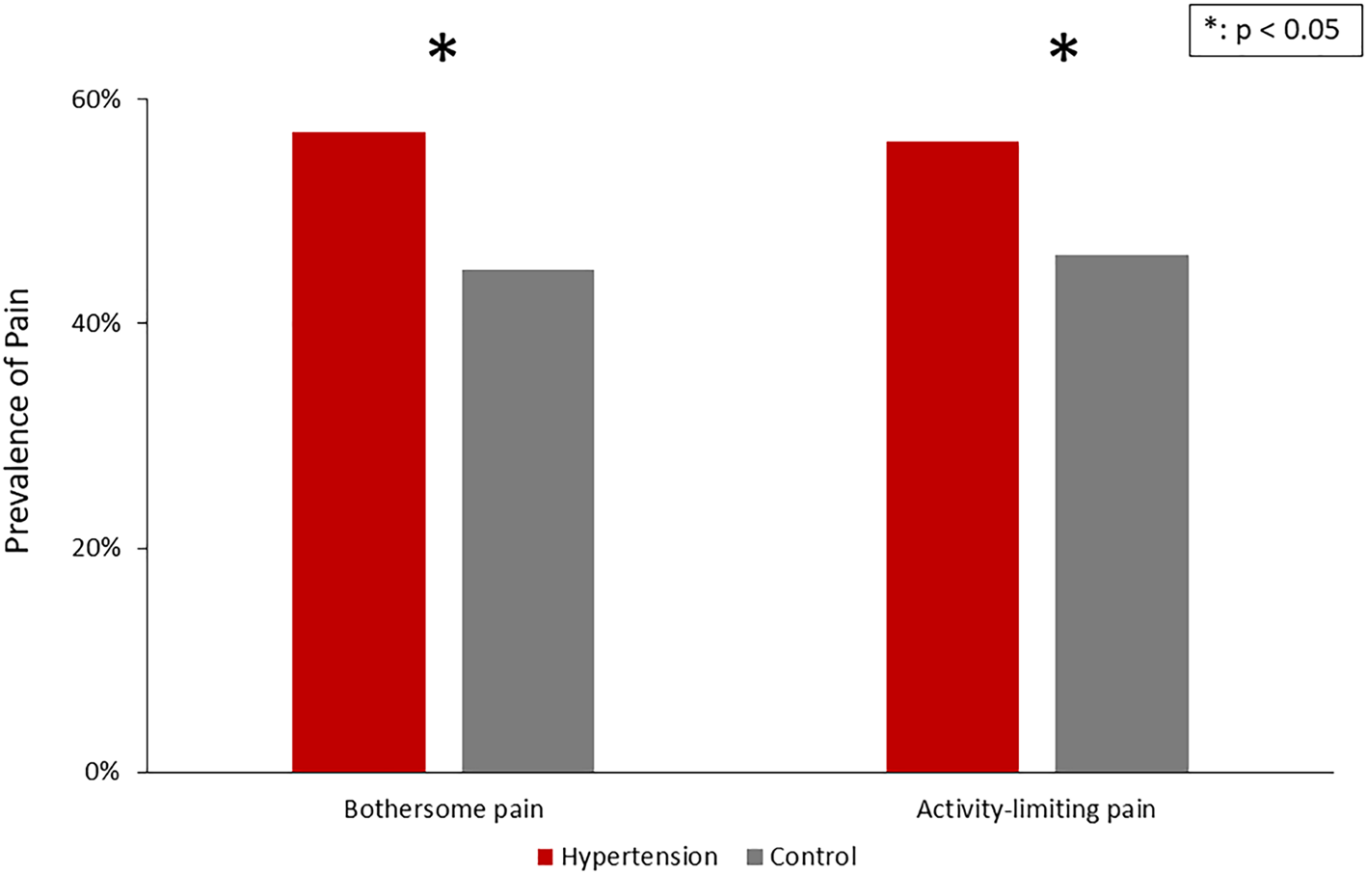
Prevalence of bothersome pain and activity-limiting pain among the hypertension and control groups. Bothersome pain and activity-limiting pain were more prevalent in the hypertension group.

**Figure 2:**
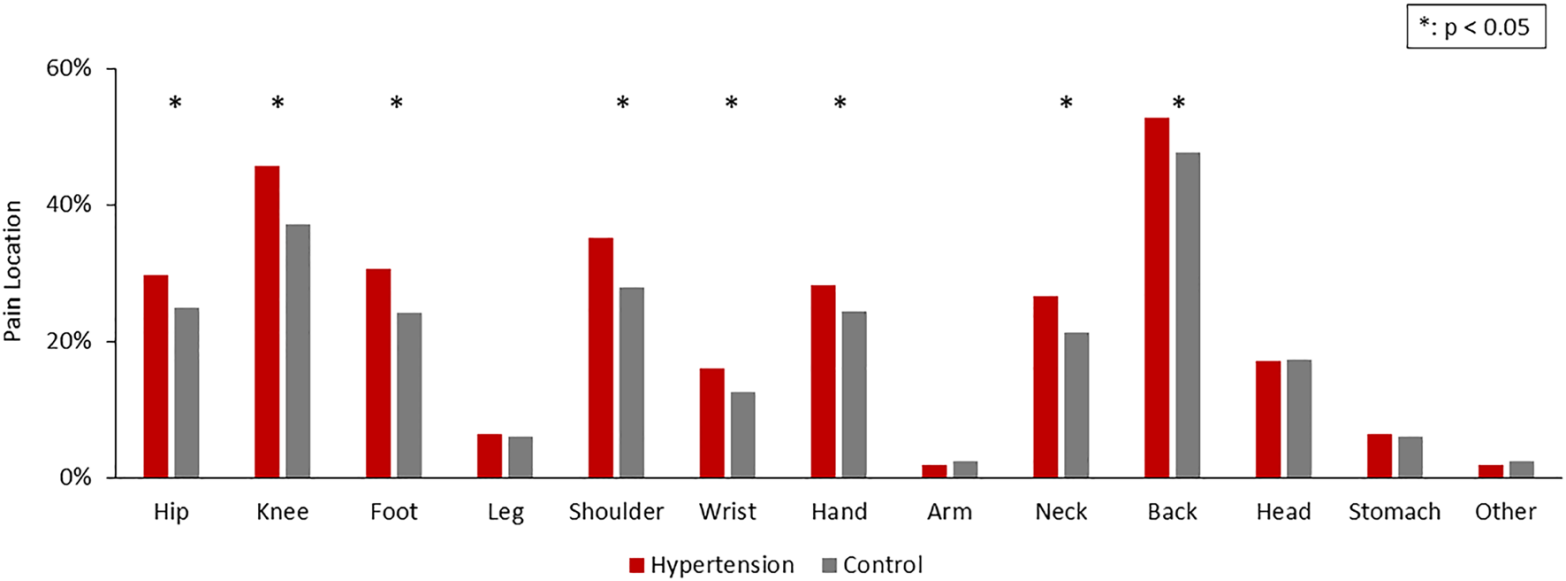
Prevalence of pain in different locations in the hypertension and control groups. Compared with the control group, the hypertension group showed more pain in most anatomical locations, mostly on the back, knee, and shoulder.

**Figure 3:**
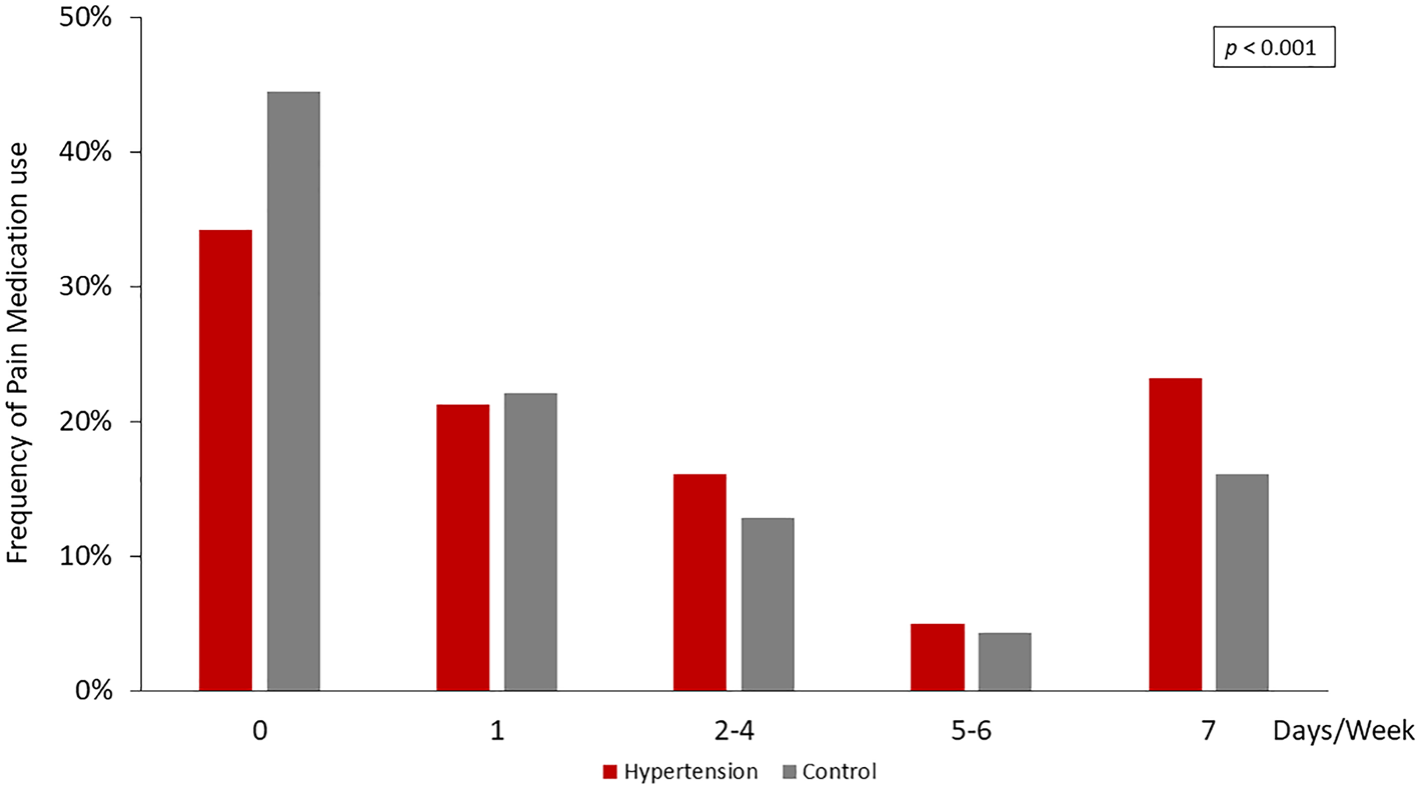
Frequency of pain medication use in the hypertension and control groups. The hypertension group had a higher frequency of pain medicine usage than that of the control group.

The hypertension group had a significantly higher prevalence of pain (57.12% vs. 44.81%, p < 0.001), activity-limiting pain (56.21% vs. 46.12%, p < 0.001) (Fig. 1), and pain discomfort among most anatomical locations (Fig. 2). The most commonly reported regions with pain included the back (52.80% vs. 47.74%, p = 0.001), knee (45.71% vs. 37.17%, p < 0.001), shoulder (35.09% vs. 27.91%, p < 0.001), and other major joints of the upper and lower limbs. In parallel, the hypertension group had a higher frequency of pain medicine usage than the control group (Fig. 3).

Table 2 shows the results of the multivariable linear mixed regression analysis for bothersome pain and activity-limiting pain. Only the covariates contained in the final models are shown in the table. After adjusting for all covariates, history of hypertension was significantly associated with activity-limiting pain (OR 1.63, 95% CI 1.06–2.52, p = 0.027), but not bothersome pain (OR 1.39, 95% CI 0.85–2.29, p = 0.185).

**Table 2:** Final model in bothersome pain and activity–limiting pain.

	Bothersome pain		Activity-limiting pain	
	Odds ratio	*p*-value	Odds ratio	*p*-value
Hypertension	1.39 (0.85–2.29)	0.185	1.63 (1.06–2.52)	0.027
**Demographic**				
Gender	1.43 (0.91–2.25)	0.118	0.90 (0.61–1.33)	0.592
Age (years old)				
65–69	Ref	Ref	Ref	Ref
70–74	1.50 (0.79–2.86)	0.219	0.84 (0.47–1.49)	0.548
75–79	1.10 (0.57–2.13)	0.774	0.78 (0.43–1.40)	0.404
80–84	0.69 (0.36–1.31)	0.253	0.40 (0.22–0.72)	0.002
85–89	0.64 (0.29–1.44)	0.282	0.60 (0.31–1.19)	0.147
≥ 90	0.45 (0.17–1.18)	0.104	0.53 (0.24–1.17)	0.115
Race/Ethnicity				
White, non-Hispanic	Ref	Ref	Ref	Ref
Black, non-Hispanic	1.72 (0.96–3.06)	0.067	0,84 (0.54–1.28)	0.415
Hispanic	0.85 (0.23–3.15)	0.806	1.93 (0.49–7.57)	0.347
Others	1.17 (0.47–2.94)	0.736	4.01 (1.41–11.38)	0.009
BMI range				
Underweight (< 18.5)	0.57 (0.20–1.61)	0.313	1.74 (0.59–5.15)	0.313
Normal (18.5–24.9)	Ref	Ref	Ref	Ref
Overweight (25.0–29.9)	1.59 (0.89–2.82)	0.115	1.22 (0.76–1.98)	0.410
Obese (≥ 30.0)	1.30 (0.76–2.24)	0.338	1.43 (0.91–2.24)	0.117
Income (≥ 28,000 USD)	0.69 (0.44–1.07)	0.099		
**Comorbidity**				
Arthritis	4.10 (2.66–6.34)	< 0.001	2.35 (1.53–3.58)	< 0.001
Osteoporosis	2.64 (1.45–4.81)	0.001		
Lung disease			1.03 (0.71–1.48)	0.891
Stroke			1.87 (1.15–3.06)	0.012
Hip fracture	2.52 (0.81–7.82)	0.109	0.64 (0.29–1.39)	0.256
Other fracture			1.61 (1.04–2.48)	0.032
**Psychological symptoms**				
Anxiety	1.81 (1.17–2.79)	0.008		
**Physical symptoms**				
Dyspnea limits activity	0.70 (0.43–1.15)	0.163	1.92 (1.31–2.83)	0.001
Fatigue limits activity	2.44 (1.42–4.22)	0.001	5.63 (3.60–8.79)	< 0.001
**Physical capacity**				
Low capacity (SPPB 0–4)	0.78 (0.43–1.44)	0.432	2.21 (1.40–3.50)	0.001
Middle capacity(SPPB 5–8)	0.58 (0.34–1.00)	0.051	1.05 (0.67–1.63)	0.830
High capacity (SPPB 9–12)	Ref	Ref	Ref	Ref
**Sleep symptoms**				
Sleep difficulty	1.60 (1.02–2.51)	0.039		

BMI: Body mass index, SPPB: Short physical performance battery.

For the other covariates, bothersome pain was significantly increased as older adults had comorbidities including arthritis (OR 4.10, 95% CI 2.66–6.34, p < 0.001), osteoporosis (OR 2.64, 95% CI 1.45–4.81, p = 0.001), anxiety (OR 1.81, 95% CI 1.17–2.79, p = 0.008), sleep difficulty (OR 1.60, 95% CI 1.02–2.51, p = 0.039), and activity-limiting fatigue (OR 2.44, 95% CI 1.42–4.22, p = 0.001). Activity-limiting pain was significantly more likely in older adults who self-reported to be a member of the other ethnic group (OR 4.01, 95% CI 1.41–11.38, p = 0.009);had comorbidities including arthritis (OR 2.35, 95% CI 1.53–3.58, p < 0.001), stroke (OR 1.87, 95% CI 1.15–3.06, p = 0.012), fracture other than hip (OR 1.61, 95% CI 1.04–2.48, p = 0.032); had physical symptoms including activity-limiting dyspnea (OR 1.92, 95% CI 1.31–2.83, p = 0.001) and activity-limiting fatigue (OR 5.63, 95% CI 3.60–8.79, p < 0.001); and low physical capacity (SPPB 0–4) (OR 2.21, 95% CI 1.40–3.50, p = 0.001).

## Discussion

The objective of this study was to assess the prevalence of pain discomfort among older individuals with hypertension in the United States. This large-scale population-based survey demonstrated that more than 50% of older Americans had experienced bothersome pain and activity-limiting pain. Among all participants, older adults with hypertension had a higher prevalence of different pain symptoms, more anatomical locations with pain, and higher frequency of pain medicine usage. After adjusting for all covariates, hypertension was significantly associated with activity-limiting pain but not with bothersome pain. These findings raise concerns about the underestimated pain comorbidities among older adults with hypertension.

Clinical studies have explored the relationship between pain discomfort and hypertension for decades, but the results remain controversial. In Hemingway’s cohort, there was no association between hypertension and low back pain^21^. Another Brazilian population study also indicated that there was no plausible association between blood pressure and musculoskeletal complaints in women and in hypertensive men not taking blood pressure medication. Although medicated men with uncontrolled hypertension may have an increased risk of musculoskeletal complaints, this could be due to the adverse effects of antihypertensive drugs rather than elevated blood pressure itself^13^. However, the Nord-Trondelag data revealed an inverse association between blood pressure and low back pain^22^. Similarly, a Korean population-based study illustrated an inverse relationship between hypertension and the prevalence of low back pain and osteoarthritis^8^. This inverse association between pain and hypertension may exist not only for musculoskeletal pain, but also for headache and migraine^7,23,24^. The inverse association between blood pressure and pain differs from our study results. The discrepancy may be due to differences in the patient populations. Most of these studies enrolled participants with younger ages (age ≥ 20 years old), while our study enrolled an older cohort (age ≥ 65 years old). Since the prevalence of pain climbs up as age increases^3^, the direction of association between hypertension and pain may be changed in older population. Also, these study participants were categorized according to blood pressure at the survey. Blood pressure measured at a specific time point may be affected by various factors of participants in the field. The lack of regular recordings may not reveal changes in blood pressure and diagnosis of hypertension.

Meanwhile, an investigation in the emergency room found that hypertension was associated with the presence of pain^25^. Another Finnish survey indicated a positive association between elevated blood pressure and the risk of low back pain^26^. Bruehl’s group revealed that chronic pain was associated with an increased risk of hypertension. Pain intensity was an independent predictor of hypertension status after adjusting for covariates^10^. In an Australian registry-based cohort, Giummarra et al. observed that the prevalence of hypertension was positively associated with persistent pain, especially with moderate to severe pain^12^. Recently, Al Ghamdi’s data revealed a significant association between hypertension and chronic pain, but not between prehypertension and chronic pain. Similarly, individuals with stage 2 hypertension were associated with a higher intensity of knee pain than that in a normotensive group^27^. These cross-sectional studies suggest that there could be a positive association between hypertension and chronic pain at the population level.

There are several potential mechanisms by which hypertension may be associated with a higher prevalence of pain. Chung’s physiological model showed that the wind-up phenomenon, a frequency-dependent increase in the excitability of nociceptive afferent neurons in the spinal cord^28^, was lower when resting blood pressure and baroreceptor sensitivity (BRS) were higher in healthy controls. The inverse association may involve the evoked descending pain inhibition pathways, attenuated ascending pain-facilitating pathways, and diminished central excitability. In contrast, the positive association between elevated systolic blood pressure and chronic pain may be associated with decreased BRS, indicating dysregulation of central pain sensitization and cardiovascular measures^29^. These results are consistent with previous research findings that impaired baroreceptor control of circulation may not only increase the risk of cardiovascular comorbidities^30^, such as hypertension, but also modulate the perception of pain^31^.

Further studies confirmed that not only BRS but also heart rate variability were reduced in people with hypertension, chronic pain, or both^32–34^. These effects suggest that the lower parasympathetic cardiovascular activity also involves the underlying mechanism of the positive association between hypertension and persistent pain.

Other theories suggest that endogenous opioid activities and neurotransmitters may play a role in the interaction between hypertension and pain. Both opioid and noradrenergic neurons are involved in central regulation of cardiovascular systems^31,35^. These neuronal activities also play a role in the modulation of C-fiber central excitability and pain sensitivity^36,37^. However, opioid blockade agents did not have an effect on pain modulation^38,39^.

Aging may contribute to the interaction between hypertension and pain. Previous studies have shown that aging is associated with reduced BRS and parasympathetic tone^30,40^, which leads to impaired regulation of blood pressure and central pain inhibition. Aging also affects the pain-modulating system. Compared to young adults, older adults showed decreased endogenous inhibition of pain sensitivity, lower threshold for temporal summation of noxious stimuli, and decreased beta-endorphin concentration and GABA synthesis^41,42^. The altered autonomic function and pain-modulating systems both predispose them to the interaction of hypertension and pain perception in older adults.

Another study suggested that the association between chronic pain and cardiovascular risk factors could act through shared risk factors, such as obesity^43^, physical inactivity, psychosocial factors, proinflammatory cytokines, and non-steroidal anti-inflammatory drug use^44^.

Similarly, frailty may participate in the relationship between hypertension and pain. Frailty, a status reflecting decline of physiological reserves and vulnerability to stress with age, is an increasing health issue among older population^45,46^. Although the components of frailty assessment scales differ from each other, fatigue and functional decline in physical, cognitive and psychosocial activities are main indicators of frailty^47^. Frailty was common among older adults with hypertension. A Meta-analysis demonstrated that the prevalence of frailty was 14% in older hypertensive people^48^. In our study, frailty symptoms, such as activity-limiting fatigue and lower physical capacities, were more prevalent among older adults with hypertension than among those without. The results indicated that frailty symptoms were positively associated with hypertension in older adults. In addition, frailty is associated with higher risk of negative health outcomes, including falls, cognitive impairment, mortality and higher health expenditure^45,46,49–51^. Consistent with other studies^52,53^, our results showed that both frailty symptoms (activity-limiting fatigue and lower physical capacities) were significantly associated with activity-limiting pain after adjusted for all covariates. Although the underlying mechanism between frailty, hypertension and pain remains unclear, it may involve endothelial dysfunction, inflammatory process and oxidative stress^47,51^.

### Strengths and limitations

Our study has several strengths. First, we used a large-scale, nationwide representative cohort of older Americans who were followed up annually by well-trained interviewers using standardized methods. The results can be generalized to almost all community-dwelling older adults in the United States. In addition, the comprehensive survey collected data on demographics, socioeconomic status, daily activities, comorbidities, and health conditions from older Americans. We included comprehensive covariates that could potentially confound the association between hypertension and pain discomfort.

This study has several limitations. First, the measurements of this study were gathered through questionnaires. Participants were asked whether they were diagnosed with “high blood pressure” instead of using physiological records from the clinicians. Similarly, bothersome pain and disabling pain were only measured by single items, while detailed pain measurements, including pain severity, duration, and origin, were not performed. However, previous studies have shown that self-reported hypertension is typically consistent with clinical diagnosis^54^ and pain symptoms used in the NHATS database have verified the questionnaire’s face validity^55–57^.

Second, although we investigated the relationship between hypertension and pain discomfort, unmeasured confounds or hidden bias could still exist because the covariates were restricted to those collected in the NHATS database. For instance, there was a lack of biomarkers data and physiological information. Also different antihypertensive drugs may affect both blood pressure and pain perception^58^. The role of polypharmacy in older people was also noted in our study, but it deserves yet further exploration. Future longitudinal research should be conducted with appropriate methods to select and control for confounding variables to explore causal relationships and underlying mechanism.

Third, this study was based on a cross-sectional analysis of a cohort study. Therefore, the causal relationship between hypertension and pain discomfort remains unestablished. Further investigation with a longitudinal design is required to elucidate the underlying correlation and mechanism. Finally, the data from the NHATS were mainly from community-dwelling adults; thus, the results may not represent older adults in residential care settings.

## Conclusion

In conclusion, community-dwelling older adults with hypertension experience a high burden of bothersome and activity-limiting pain. After adjusting for covariates, hypertension remained independently associated with activity-limiting pain. Therefore, it is important for clinicians and researchers to provide appropriate assessments and treatments for older adults with both hypertension and pain. Further studies, including biomarkers and physiological data, may have a longitudinal design to improve our understanding of the potential mechanisms involved.

## Data Availability

The data that support the findings of this study are openly available in the National Health and Aging Trends Study (NHATS) at https://nhats.org/researcher/data-access/public-use-files, reference number Grant number NIA U01AG032947. NHATS offers registered users three types of files: downloadable NHATS public data files, downloadable NSOC and other sensitive data files, and restricted files. All files require registration with a username and a password. Sensitive and restricted files require an additional application.
